# Ankle fractures in patients over age 55 years: Predictors of functional outcome

**DOI:** 10.1097/OI9.0000000000000080

**Published:** 2020-07-27

**Authors:** Natasha M. Simske, Alex Benedick, Megan A. Audet, Heather A. Vallier

**Affiliations:** MetroHealth Medical Center, affiliated with Case Western Reserve University, Cleveland, Ohio.

**Keywords:** ankle fracture, complications, elderly, functional outcomes, geriatric

## Abstract

**Objectives:**

To identify predictors of functional outcomes following treatment of ankle fracture in patients 55 years or older.

**Setting:**

Level 1 Trauma Center.

**Patients/participants:**

Four hundred twenty-nine patients with torsional ankle fractures (44A-C): 233 patients (54%) were ages 55 to 64, 25% were between the ages 65 and 74; 21% were 75 years or older.

**Intervention:**

Operative or nonoperative management of ankle fracture.

**Main outcome measure:**

Early complications were assessed for all patients after minimum of 6 months, and functional outcome scores as assessed by the Foot Function Index (FFI; n = 166, 39%) and Short Musculoskeletal Function Assessment (SMFA; n = 168, 39%) after median 57 months follow-up.

**Results:**

Surgical management was elected in 67% of patients. Nonoperative management became more common with advancing age and was associated with fewer unplanned operations (12% vs 3%, *P* < .01) and complications (21% vs 13%, *P* = .07). African American race was associated with worse pain on the FFI (*P* = .002) and BMI was associated with worse (higher) scores on all categories of the FFI and SMFA (all *P* < .05). Diabetes, neuropathy, and mental illness were also predictive of worse scores on various categories of both surveys. Assistive device use or nonambulatory status at the time of injury was associated with worse disability/dysfunction, activity, and mobility scores on both the FFI and SMFA (all *P* > 15, *P* < .05). Sex, Hispanic ethnicity, tobacco use, open fracture, dislocation, fracture pattern, and operative management were not independent predictors in this regression model.

**Conclusions:**

Baseline health and ambulatory capacity at injury were more predictive of outcomes following ankle fracture than were fracture characteristics or type of treatment.

## 1 Introduction

Ankle fractures are the third most common fracture among the elderly.^[1]^ As the population ages, ankle fractures in geriatric populations will become more widespread, with open injuries as a result of low-energy mechanisms making up a large proportion of these.^[2–4]^ Geriatric ankle fractures are associated with better survivorship and fewer complications than hip fractures and other hospital admissions in the elderly.^[5–7]^ However, fracture management and rehabilitation are often confounded by several factors in this population. Higher rates of medical comorbidities place elderly patients at risk for complications, and older patients often have limited social support and poor physical health that may be obstacles to recovery of preinjury function.^[6–8]^

Fixation of unstable, displaced ankle fractures is well supported, with some evidence that it may also be the ideal treatment modality for middle-aged to elderly patients.^[9–13]^ However, nonoperative management produces acceptable outcomes as well, with minimal subsequent arthrosis and dysfunction, if satisfactory closed reduction can be obtained.^[14–18]^ Older age, and presence of diabetes or peripheral vascular disease are conditions that may lead to recommendation of nonoperative management.^[19,20]^ In a few randomized controlled trials comparing open reduction and internal fixation with nonoperative management, better functional outcomes have not consistently followed 1 treatment modality.^[18,21–24]^

Although functional outcomes have been well explored in operatively-managed ankle fracture populations, there has been a limited investigation of potential determinants of dysfunction following ankle fracture in elderly populations.^[25–31]^ Age has been evidenced as a predictor of poor long-term functional outcomes, but the factors that influence outcomes may change with aging as middle-aged and elderly patients have different lifestyles and activity restrictions than their younger counterparts.^[32]^ This study will identify factors that contribute to worse functional outcome and will specifically assess whether nonoperative management plays a role in this relationship.

## 2 Patients and methods

### 2.1 Data collection and variables of interest

Following Institutional Review Board approval, all patients 55 years or older who sustained a torsional ankle fracture (AO/OTA 44A-C) between 2006 and 2015 were identified.^[33]^ Four-hundred twenty-nine patients met such criteria. Research was conducted in accordance with the Declaration of the World Medical Association and informed consent was obtained as required. Charts and radiographs were reviewed for basic demographics, presence of medical comorbidities, use of assistive devices, and ambulatory status prior to injury. Mechanisms of injury, fracture pattern, and management were also obtained.

Nonoperative fractures were divided into 2 groups: “stable” patterns including 44A1.2, A1.3, and B1.1, or those with mortise stability as indicated by the treating surgeon and “unstable” patterns including those with an associated dislocation, unstable proximal fibular fracture, syndesmotic injury, and/or posterior malleolus fracture. Final alignment of unstable, nonoperative fractures was determined based on radiographs taken after bony healing. Alignment was categorized as *anatomic*: anatomic ankle mortise, less than 2 mm of fracture displacement and no subluxation on radiography; *adequate*: anatomic ankle mortise, with ≥2 mm of fracture displacement, and no subluxation on radiography; *displaced*: talus retained in ankle mortise with mortise widening and/or angular displacement of the ankle joint 5 or more degrees from the normal mechanical axis; *poor*: talus subluxation from the ankle mortise.

Postoperative complications were recorded, including nonunion, malunion, superficial infection, and deep infection. Infections were either superficial, treated on an outpatient basis with local wound care and oral antibiotics; or deep, requiring surgical debridement and irrigation and intravenous antibiotics. Any wound-healing complications that required additional wound care were similarly recorded. Malunions were described as >5° in any plane and/or residual medial clear space or syndesmotic widening, and nonunions were defined as an absence of callus formation or other evidence of healing after 6 months. Secondary procedures for complications or otherwise related to the injured ankle were recorded.

After a minimum of 12 months following injury, functional outcomes were assessed. The Foot Function Index (FFI) was utilized for lower extremity-specific outcomes and the Short Musculoskeletal Function Assessment (SMFA) was used to assess generalized musculoskeletal outcomes.^[34–37]^ All patients were contacted on up to 3 occasions to complete surveys, either by mail or over the phone. Changes to baseline ambulatory status were documented from the medical record and/or based on survey responses.

### 2.2 Treatment

Ankle fractures were either treated surgically or nonoperatively. Standard techniques of open reduction and internal fixation were used for all operatively-managed fractures, with surgical timing and technique at the discretion of the treating surgeon. Nonoperatively managed fractures were treated with closed reduction and casting. Timing of weightbearing was determined by the treating surgeon, depending on the fracture pattern. In the case of nonoperative management, initial reduction quality was either anatomic or adequate in all cases. Open fractures were treated with urgent surgical debridement followed by open reduction and internal fixation using small fragment and/or mini fragment stainless steel implants. Of the 79 open fractures, there were 3 cases in which patients were managed with external fixation (n = 2) or closed reduction prior to wound closure (n = 1). All patients were splinted postoperatively, and a period of nonweightbearing and elevation were initially recommended. Based on fracture pattern and clinical and radiographic evidence of healing, weightbearing was deferred for 6 to 12 weeks following surgery.

### 2.3 Statistical analysis

Demographics, medical comorbidities, injury characteristics, postoperative complications, unplanned secondary procedures, and functional outcomes data were independently compared for age groups: 55 to 64 years, 65 to 74 years, and 75 or more years, and between nonoperative and operative populations. Chi-squared tests (including Fisher exact test, when indicated), one-way analysis of variance or Student *t* tests were used to compare these groups. Multiple regression was performed to investigate the relationship between patient reported outcomes (as measured by the FFI and SMFA) and patient demographics (age, sex, race), medical history (BMI, diabetes, neuropathy, renal disease, psychiatric history, tobacco use), ankle injury characteristics (fracture dislocation, open fracture, OTA classification), management (ankle surgery), and preinjury ambulatory status. In all instances statistical significance was set to *P* < .05.

## 3 Results

### 3.1 Demographics and medical comorbidities

Four hundred twenty-nine patients age 55 years or older sustained ankle fractures over a 9-year period. One hundred sixty-two patients (38%) were male and 75% were Caucasian, with an average BMI of 32 (Table 1). Eighty-five percent of patients had at least 1 comorbid medical condition present at the time of injury, with obesity (BMI > 30, 51%), diabetes (34%), psychiatric illness (21%), and pulmonary conditions (10%) being most common. Patients who were 75 years or older were more likely female, had more medical comorbidities, and less substance use (tobacco, alcohol, and illicit drugs) when compared with their counterparts under age 65.

**Table 1:**
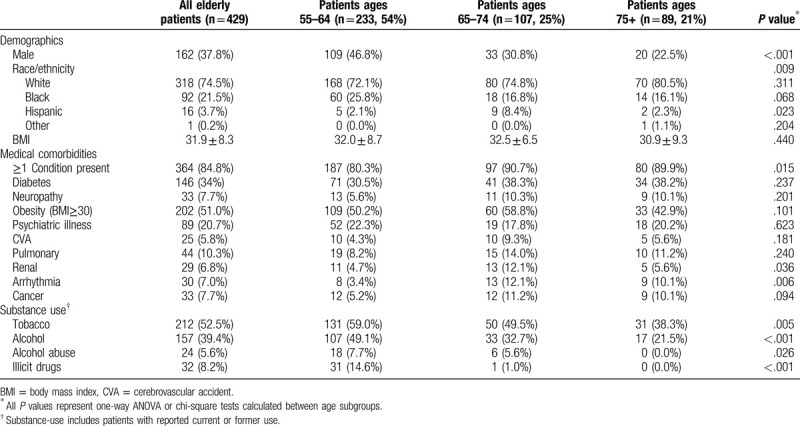
Demographic information, medical comorbidities, and substance use.

### 3.2 Injury details and fracture characteristics

Most injuries (71%) were sustained after a low energy fall (Table 2). Seventy-nine patients (18%) had open injuries due to medial wounds occurring at the time of dislocation, and most were Weber B fractures (74%). No specific fracture classification or mechanism of injury was associated with a particular age group. However, patients over age 75 had fewer associated deltoid ligament injuries (11%) and more medial malleolus fractures (72%) when compared with patients ages 55 to 64, both *P* < .05. A deltoid injury was defined as medial soft tissue injury with associated displacement evidenced by a wide medial clear space on plain radiographs. Operative treatment was undertaken in 67% of patients. Nonoperative management became more common with advanced age (45% in patients over age 75 vs 27% in patients ages 55–64, *P*≤.005).

**Table 2:**
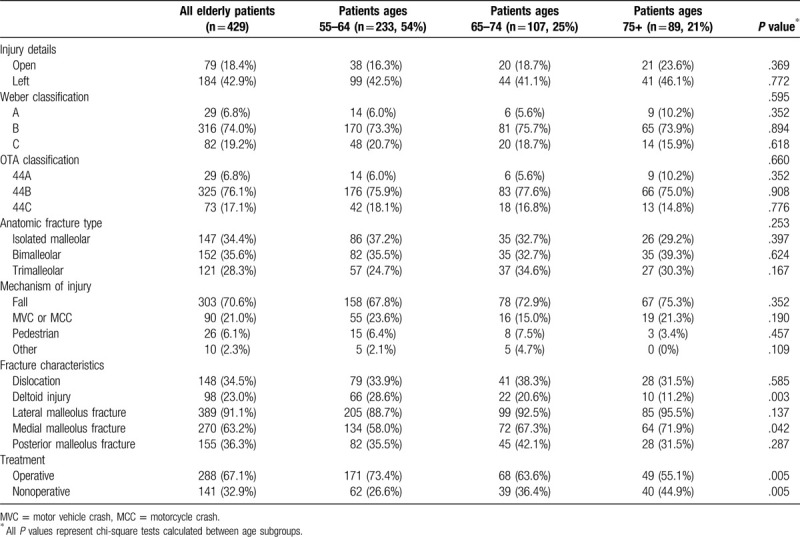
Injury information including mechanism of injury, fracture patterns, and the presence of open fractures and other injuries.

### 3.3 Ambulatory status and use of assistive devices

The majority of patients (n = 295, 70%) were ambulatory at the time of injury, with no consistent use of assistive devices for ambulation (Table 3). Rates of preinjury assistive devices for ambulation were significantly higher with advancing age (15% among patients 55–64 years; 28% among patients 65–74 years, and 38% among patients 75+, *P* < .01). After full recovery, 91 patients (21%) had a change in their ambulatory status, with new use of assistive devices or becoming nonambulatory. More often, the older patients required permanent postinjury use of an assistive device (54% for those over age 74, and 50% in those ages 65–74 years vs 33% in patients 55–64 years old), *P* < .001.

**Table 3:**
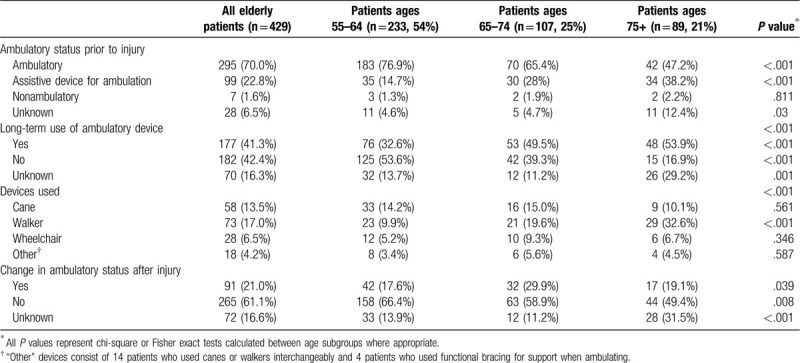
Ambulatory status and device use prior to and following injury.

### 3.4 Operative vs nonoperative management

There were 80 postinjury complications (19%) and 39 patients who required secondary operations (9%) (Table 4), mostly implant removal. Patients who underwent nonoperative management had fewer subsequent procedures (12% vs 3%, *P* < .01). Although the surgical population had a trend for an overall higher rate of complications (21% vs 13%, *P* = .065), no difference was seen after removing open fractures (14% vs 13%). More superficial infections and wound-healing problems associated with operatively managed fractures were largely attributed to open fracture etiology. Considering only malunions and nonunions to make a more direct comparison between surgical and nonsurgical patients regarding complications, with the numbers available no significant differences were identified, only a trend for more malunion in nonoperative patients (4.8% vs 1.7%, *P* = .065).

**Table 4:**
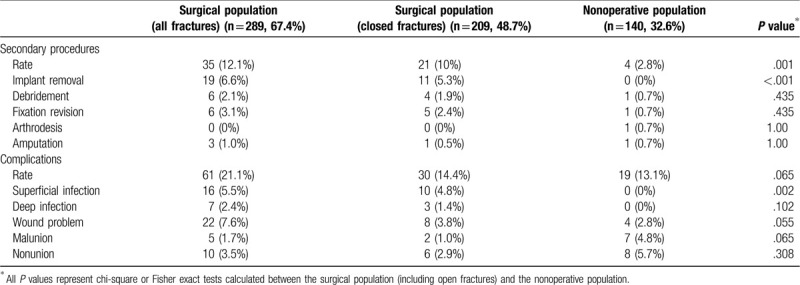
Rates of complications and unplanned operations among the geriatric population based on treatment modality: surgical management vs nonoperative/closed treatment.

Of the 140 patients with nonoperative ankle fractures, 64 patients (46%) had mechanically stable fracture patterns (all 44A1 or 44B1). The remaining patients had unstable fracture patterns managed nonoperatively following satisfactory closed reduction or due to the patient's health status, possible substance use or psychiatric disorders, or anticipated noncompliance. Patients with mechanically stable fractures had no additional operations and 2 complications (3.1%), both asymptomatic nonunions, compared with 4 additional procedures (5.1%) and 17 complications (21.8%) among patients with unstable fracture patterns. Among patients with unstable patterns, 32% had anatomic final alignment, 24% were adequate, 35% were displaced, and 10% were poor. Five patients were excluded from this analysis due to insufficient radiographic follow-up.

### 3.5 Functional outcomes

After minimum 12 months following injury, 39% of patients completed outcomes surveys (FFI: n = 166; SMFA: n = 168). Twenty-three patients (6%) were known to be deceased and were unavailable to complete surveys (Table 5). Response rates were lower for those over age 74 (18% vs 44%, *P* < .001). Surgically managed patients were more likely to complete outcome surveys, with a response rate >10% higher on both surveys (*P* < .05 for both). However, there were no differences in mean scores on any subindices of either the FFI or SMFA. This finding remained unchanged after removing patients who sustained open fractures. Median time to survey was 57 months for the FFI and 58 months for the SMFA (range: 19–133 for both). Time to survey was no different between age groups. Univariate comparisons showed no significant differences in outcome scores except for the worse activity subscore of the FFI in those age 75+ years (41 vs 22 in patients 55–64 years), *P* = .03. Patients with unstable patterns managed nonoperatively also had worse functional outcomes. Although FFI scores did not differ significantly between groups, all SMFA subscores were significantly higher for patients with unstable patterns, excluding the Arm and Hand subscore (all *P* < .05).

**Table 5:**
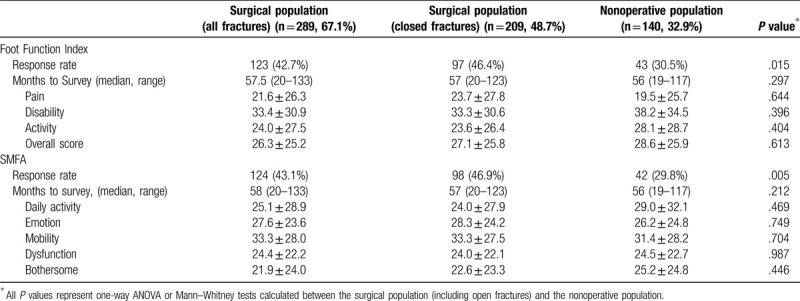
Patient-reported outcome measures (PROMs) as measured with short Musculoskeletal Function Assessment and Foot Function Index surveys.

Multiple linear regression identified several independent predictors of worse functional outcome scores, most notably BMI on all subindices of both surveys (Table 6). Prior use of assistive devices and nonambulatory status at the time of injury were also associated with worse outcomes. Age was only linked with worse outcomes on the daily activity subindices of the SMFA (*P* = .036). Other significant predictors on at least one category of either the FFI or SMFA included African American race, diabetes mellitus, neuropathy, and a positive psychiatric history. Interestingly, renal disease (chronic kidney disease or end stage renal disease) was associated with better (lower) scores on the activity category of the FFI (*P* = .024). Open fracture, fracture dislocation, fracture pattern, or operative management were not predictive of better or worse scores on any subindices of either survey.

**Table 6:**
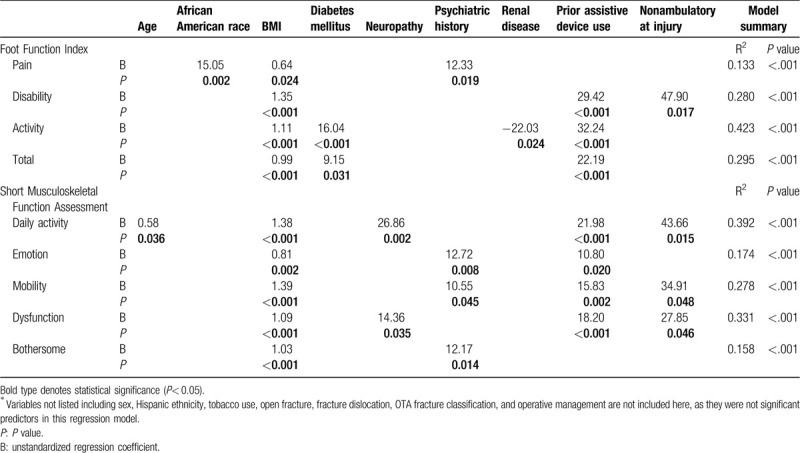
Results of multiple linear stepwise regression using patient demographics, medical history, and injury characteristics to predict patient reported outcome scores, as measured by the Foot Function Index and Short Musculoskeletal Function Assessment surveys.^∗^

## 4 Discussion

This retrospective study indicated that higher BMI, various medical comorbidities (diabetes, neuropathy, and mental illness), as well as hindered ambulation at the time of injury were all predictive of worse outcomes (higher scores) on at least 1 category of either the FFI or SMFA. However, fracture characteristics including open injury, dislocation, fracture pattern, as well as surgical management were not associated with either better or worse reported outcomes. To the author's knowledge, this is the largest study of operatively and nonoperatively managed ankle fractures in middle-aged and geriatric populations to assess for possible determiners of poor functional outcomes.

Nonoperative management of ankle fractures vs operative treatment has long been a point of debate, with most surgeons opting for definitive fixation for unstable or displaced fractures. Evidence shows that surgical treatment leads to optimal results and several randomized controlled trials have observed improved or noninferior functional outcomes when compared with nonoperative management.^[9–12,21–24]^ Still, nonoperative treatment appears satisfactory and potentially appropriate for some patients and may achieve outcomes that are consistent over time.^[14–18]^ However, in a large study looking at Part A inpatient claims from a Medicare database, nonoperative management was associated with a higher odds ratio of death within 1-year of injury and a mortality rate >10% higher than operatively managed fractures.^[5]^ Given that predictors of nonoperative management include older age, higher Charlson Comorbidity Index scores, and presence of diabetes or peripheral vascular disease, this high mortality rate may be more a function of poor health at baseline vs the elected treatment option.^[13,19]^ It is clear that baseline health and ability to restore preinjury function are important to consider when discussing options with patients and family members.

Several studies have found younger age,^[20,25–31]^ male sex,^[26,27]^ American Society of Anesthesiologists score of 1 or 2^[26,28]^ absence of diabetes,^[26,38]^ and syndesmosis reduction^[27,30,32]^ to be linked with better functional outcomes scores on questionnaires both extremity-specific and generalized measures. Although some studies have documented more baseline dysfunction among elderly patients, most acknowledge that once these differences are accounted for, younger and older patients have similar outcomes.^[25,31]^ Our findings agree with some existing studies evaluating patients of all ages. For example, we found that patients who were unhealthier at baseline, with higher BMI, diabetes mellitus, or neuropathy had worse outcomes. Psychiatric illness was also associated with worse scores on the pain category of the FFI and on the emotion, mobility, and bothersome categories of the SMFA, consistent with prior work.^[39]^

At time of injury, our population had a high frequency of ambulatory limitations. Furthermore, use of a walker to ambulate in the community postinjury became more widespread with advanced age. Not surprisingly, use of assistive devices at time of injury was associated with worse outcomes scores on the FFI and the bothersome category of the SMFA. Being nonambulatory at time of injury was also linked with greater disability/dysfunction on both the FFI and SMFA, as well as worse mobility and daily activity scores on the SMFA. Although these findings are to be expected, it is important that they were controlled for in our regression model. All other variables that were found to be predictive of worse functional outcomes were independent of ambulatory status or assistive device use.

Strengths of this study include analysis of a large sample of patients with common injuries and a group that is increasing over time due to population growth and longer life span. The retrospective design of this study introduces a number of limitations. Medical comorbidities and ambulatory deficiencies may have been underreported. Another limitation was the inability to collect questionnaires from all patients. However, a large number of patients (39%) still completed surveys, though there was a clear pattern toward younger patients (<75 years) more likely to respond. This is consistent with adult lifespan and greater likelihood of some patients deceased within the follow-up period, unrelated to the ankle fracture per se. In general, only capturing a fraction of the total number of patients in the study introduces sampling bias, in which patients with worse function, mobility, or pain may be more or even less likely to complete questionnaires. Our study is further limited by lack of indications for treatment, which was at surgeon and patient discretion. Additionally, better reduction of the syndesmosis and earlier weightbearing have been associated with improved outcomes after operative and nonoperative treatment for ankle fracture.^[9,27,30,32]^ We further did not determine quality of reduction and final alignment by computed tomography scans. One could argue that surgically managed fractures probably achieved better quality reductions. Assuming this to be true, surgical treatment was not an independent predictor of better or worse functional outcome scores. Therefore, the authors posit that in elderly patients, baseline health and activity may be more indicative of long-term outcomes, vs achieving superior reduction quality.

Patients 55 years or older with higher BMI, concomitant diabetes or neuropathy, mental illness and use of assistive devices for ambulation are at increased risk for poor functional outcomes following ankle fracture regardless of type of treatment. Details related to injury including open fracture, dislocation, fracture pattern, and operative management were not significant predictors in our regression model. It is important to consider a patient's baseline quality of life and activity capacity prior to treatment, seeing as these factors have more influence over functional outcomes than operative versus nonoperative treatment in patients with baseline debility.
